# Enhancing dialogue: Introducing ‘Letters to the Editor’

**DOI:** 10.1007/s12471-024-01864-w

**Published:** 2024-03-19

**Authors:** Pim van der Harst

**Affiliations:** Department of Cardiology, Division of Heart & Lungs, University Medical Center Utrecht, Utrecht, The Netherlands

In this issue we introduce a further enhancement to the journal. Our objective extends beyond just presenting original research; we also strive to nurture a dynamic, interactive dialogue among our readers. To achieve this, we are introducing a new (or updated) article category, ‘Letters to the Editor’. The purpose of this category is to encourage concise, yet incisive, critiques of our recently published articles. Contributors must adhere to a 175-word limit with a maximum of three references, and refrain from including figures or tables, to ensure a focused and impactful initiation of dialogue. The original article’s authors will than have the chance to reply, thus enriching the exchange of ideas.

We believe this initiative will further engage our readership and contribute to the ongoing improvement of cardiovascular knowledge. This issue exemplifies this approach, with Minten et al. not only demonstrating NHJ’s influence beyond the Netherlands, but also engaging with a recent article [1] and offering insightful remarks on the ideal timing of PCI and the role of coronary physiology in patients undergoing TAVI implantation [2].

While space constraints may limit the number of letters we can feature in print, we are exploring digital means to involve a wider participation. These efforts are in line with our commitment to the Netherlands Society of Cardiology and our goal to intensify reader involvement.

I am excited to be developing a more collaborative and discussion-rich academic setting in our journal. Your critical insights are not only welcome but vital for a vibrant exchange of ideas that moves the cardiovascular field forward.
